# Unmet Need for Family Planning and Spousal Separation in Nepal: A Spatial and Multilevel Analysis

**DOI:** 10.3389/ijph.2023.1606395

**Published:** 2023-12-07

**Authors:** Yoona Kim, Zoé Mistrale Hendrickson, Manju Shakya, Young Su Park, Myunggu Jung

**Affiliations:** ^1^ Moon Soul Graduate School of Future Strategy, College of Liberal Arts and Convergence Science, Korea Advanced Institute of Science and Technology, Daejeon, Republic of Korea; ^2^ Bloomberg School of Public Health, Johns Hopkins University, Baltimore, MD, United States; ^3^ International Development Studies, Hankuk University of Foreign Studies, Seoul, Republic of Korea; ^4^ Department of the History of Medicine and Medical Humanities, College of Medicine, Seoul National University, Seoul, Republic of Korea

**Keywords:** migration, Nepal, reproductive health, spousal separation, unmet need for family planning

## Abstract

**Objectives:** In Nepal, where increasing numbers of married couples live apart due to migration, progress in reducing unmet need for family planning (UMN) is stagnant. This study aims to identify spatial patterns of UMN of married women and spousal separation in Nepal and explore associations between UMN and spousal separation at individual- and district-levels.

**Methods:** We used 2016 Nepal Demographic and Health Surveys data to conduct spatial and multilevel logistic analyses.

**Results:** This study shows evidence of similar geographical patterns in UMN of married women and spousal separation. At the individual level, women living with their spouses had 88% (aOR = 0.12, 95% CI 0.11–0.13) decreased odds of experiencing UMN compared to those living apart from their spouses. While not statistically significant, increasing odds of UMN were observed with higher prevalence of spousal separation at the district level.

**Conclusion:** This study contributes to the existing literature by showing similar geographical patterns of UMN and spousal separation across Nepal and demonstrating both individual and contextual effects of spousal separation on UMN among married women. Theoretical and policy implications are discussed.

## Introduction

Unmet need for family planning (UMN) occurs when women desire not to become pregnant but do not use a method to prevent pregnancy [1]. UMN can result in poor maternal and child health outcomes including unsafe abortions, maternal deaths, and high infant mortality [2, 3]. In Nepal, despite the government’s efforts to make contraceptive methods accessible, prevalence of modern contraceptive use and UMN plateaued among currently married women aged 15–49. The prevalence of modern contraceptive use was 44% in 2006 and 43% in 2016, while UMN also barely changed from 25% in 2006 to 24% in 2016 [4–6].

Among numerous factors from women’s age, number of previous children, and level of education to gender dynamics, spousal separation was highlighted as an influential factor associated with modern contraceptive use and UMN in countries like Nepal where labor out-migration is prevalent [7–11]. The stagnant progress in increasing uptake of modern contraceptives and reducing UMN in Nepal has been attributed to a high percentage of spousal separation due to male out-migration [4]. Over the past decade, overseas employment has become prevalent in Nepal. More than 1.1 million foreign labor approvals were issued between 2019 and 2022, a figure that represents around 15% of the total labor force [12, 13]. Considering both internal labor migration within Nepal and labor migration to India (not classified as international migration due to the open border), the number of migrants is believed to be significantly higher [12]. With the increasing number of labor out-migration from Nepal, spousal separation is expected to become more influential. In 2016, 34% of currently married women reported their husband lives away from home [6].

Use of family planning is influenced not only by individual socioeconomic conditions, but also by community-level factors including geographic location and availability of reproductive health services in the community [4, 8, 14, 15]. A study that compared low and high male out-migration locations in India found that women with migrant husbands were less likely to use modern contraceptives if they resided in high out-migration areas compared to low [8]. Similarly, how common spousal separation is in a community may be associated with UMN in the community through changes in household income, social norms for accessing family planning services, and female autonomy [16–20]. Remittance from spouses working overseas can increase overall household income, which has been linked to reduced UMN [11, 16, 17]. On the other hand, separation with migrant spouses may restrict access to reproductive health services through stricter social control of wives living without their husbands [9, 18, 19]. However, existing studies about UMN among women with migrant spouses in Nepal focus on individual and household-level factors [7, 15, 21].

To understand individual- and community-level pathways linking spousal separation and UMN, this study draws from theories about fertility behavior. Advocates of the adaptationist approach argue that fertility behavior primarily responds to socioeconomic conditions, which change the costs and benefits associated with childbearing. In contrast, the diffusionist hypothesis argues cultural values not only influence individual couples’ fertility behaviors, but those of others around them regardless of their socioeconomic conditions [17]. The diffusionist approach underscores the contextual effects of community-level migration patterns on reproductive health behaviours in the community. In Mahapatra and Saggurti's study [8], the authors explained the contextual effect of regional prevalence and patterns of male out-migration with community healthcare workers not perceiving wives of migrant workers as needing family planning services and diminished contact with healthcare workers as a result. The study also found social stigma against wives of migrant workers accessing family planning services in physical absence of their husbands. Women’s intention to use family planning services differed by how far and how long the migrant husbands are away [8].

Both theories and empirical findings support the view that UMN among women of reproductive age is associated with individuals’ socioeconomic conditions as well as community-level contexts and migration patterns. However, little is known how UMN of married women in Nepal, where spousal separation is prevalent due to their husband’s labor migration, is associated with individual and community factors. Therefore, this study aims to examine a) spatial patterns in UMN and spousal separation of married women in Nepal and explore b) the association between UMN and spousal separation of married women at the individual- and district-levels. This study hypothesizes that a) UMN and spousal separation exhibit similar spatial patterns and b) spousal separation both at the individual- and district-levels will be associated with higher UMN in Nepal. The results can inform resource allocation of family planning programs and policies that address UMN among both married couples living apart and in areas with higher spousal separation. Public health programs based in contexts of high spousal separation can facilitate meeting targets for universal access to sexual and reproductive healthcare services in Nepal, aligning with the Sustainable Development Goals (3.4 and 5.6) and the Nepal government’s Right to Safe motherhood and Reproductive Health Act 2018 [22].

## Methods

### Data

We used data from the 2016 Nepal Demographic and Health Surveys (NDHS) downloaded from the DHS website after explicit permission granted by the data originators [6]. The 2016 NDHS includes information about family planning and reproductive health of 12,862 women of reproductive age (15–49 years). Among the total sample (*n* = 12,862), we filtered married women (*n* = 9,897) as the primary interest of this study is to explore the association between UMN and spousal separation. In addition to the women data, the DHS also provides household data that contains basic demographic information of each household member in selected households, including migration status. The women data were merged with household data to obtain a complete dataset required for the analysis.

The 2016 NDHS sample was stratified and selected in two stages (wards and households) in rural areas and three stages (wards, Enumeration Areas, and households) in urban areas. From the selected households, all women aged between 15 and 49 who slept in the house the night before the survey were eligible to complete the questionnaire designed for women. We also obtained Global Positioning Systems coordinates for 383 wards (Primary Sampling Units; PSU) through the NDHS programme to create a high-resolution spatial map of spousal separation and UMN across the country. Administrative unit shapefiles were obtained through the freely available Database of Global Administrative Units through the DIVA-GIS project [23].

### Variables

#### Outcome Variable

The outcome variable for this study was the binary variable of unmet need for family planning (UMN) comparing those with an unmet need to those with no unmet need. Married women of 15–49 years were defined as having an unmet need for family planning when they 1) were fecund, 2) reported not wanting any (more) children, wanting to delay the birth of child for at least 2 years, or being undecided about the timing of the next birth, and 3) were not using any method of contraception [24].

#### Explanatory Variables

The main explanatory variables were spousal separation at the individual- and district-level. Spousal separation at the individual level was determined by the item asking women whether they were currently residing with husband/partner or not. Duration of spousal separation compared those separated for less than 1 year to those separated for 1 year or longer. Community prevalence of spousal separation was calculated as the percentage of women in each district that were currently living separately from their spouse.

#### Individual- and Household-Level Variables

Covariates at the individual level included socio-demographic variables such as women’s age (<25, 25–34, 35–49), women’s highest level of education attained (less than primary, primary, secondary, and higher education), as well as measures of women’s reproductive history including experience of an abortion (yes/no) and number of living children (0, 1, 2, and more than 2). We also used household-level variables such as spouse’s highest level of education attained (less than primary, primary, secondary, and higher education), caste/ethnicity (Brahmin&Chhetri, Dalit, Janajati, Newar, Muslim, and Other), household wealth (poor, middle, and rich), and number of household members (<4, 4, 5, and >5). Caste/ethnicity followed recommended categorization from previous NDHS and combines caste groups (Brahmin&Chhetri, Dalit), indigenous ethnic groups (Newar, Janajati), and others [25]. Household members were those who lived together in the same dwelling unit, acknowledged one adult as the head of the household, shared the same housekeeping arrangements, and are considered as one unit [26].

#### District-Level Variables

Covariates at the district level were ecological zone (hill, mountain, and terai), urbanicity (urban/rural), province, and access to healthcare, calculated by the mean duration to the nearest healthcare facility by district.

### Data Analysis

#### Spatial Autocorrelation of UMN and Spousal Separation at the Primary Sampling Unit Level

We first estimated UMN and spousal separation at the PSU level and assessed both the global and the local spatial autocorrelation of UMN and spousal separation at the PSU level by using the Moran’s I and the local indicator of spatial association (LISA). The inverse distance weighting (IDW) was applied to explore the spatial patterns underlying UMN and spousal separation prevalence across the country. The global Moran’s I value ranges from −1 to +1, with a positive value indicating positive spatial autocorrelation (i.e., the presence of similar values at neighbouring locations) and a negative value indicating negative spatial autocorrelation (i.e., the presence of dissimilar values at neighbouring locations). Moreover, we used the LISA map to display contiguous PSUs with similar UMN prevalence or percentage of spousal separation to their neighbouring PSUs as two possible types, “high–high” and “low–low” areas.

#### Geospatial Regression Modelling of UMN and Spousal Separation

To predict the prevalence of UMN and spousal separation across Nepal, we used the Bayesian geostatistical modelling framework. Conditional on the true prevalence $P\left(x_{i}\right)$ of UMN or spousal separation at location $x_{i},i=1,\ldots ,n,$ we assumed that the number of cases $Y_{i}$ out of $N_{i}$ women sampled follow a binomial distribution: $Y_{i}\left|P\right.\left(x_{i}\right)∼Binomial\;\left(N_{i},P\left(x_{i}\right)\right)$ . Then, mean predicted prevalence of UMN and percentage of spousal separation was modelled via a logit link function to a linear predictor defined as:

$$logit\left(P\left(x_{i}\right)\right)=a+S\left(x_{i}\right)$$

where α is the intercept and $S\left(\cdot \right)$ is spatial random effects modelled using a zero-mean Gaussian Markov random field with a Matérn covariance function. Technical details of the model and model building process are given in the Supplementary Appendix. To show uncertainty related to the prediction, we presented the spatial distribution of credible intervals by calculating the differences between 5% and 95% percentiles of the predicted UMN and spousal separation across the country.

#### Multilevel Analysis

To assess the statistical associations between the outcome (UMN) and explanatory variables, we first examined the distribution of respondents by status of spousal separation using relative frequencies. Pearson’s chi-square tests were used to test for significance. Second, a two-level mixed effects logistic regression analysis was conducted to estimate adjusted odds ratios (aORs) and the degree of random variations between districts. Specifically, four models were fit. Model 0 (null model) included the outcome variable to assess the variance in UMN between districts and to estimate the intraclass correlation coefficient (ICC). Model 1 consisted of the outcome variable and individual level factors, Model 2 consisted of the outcome variable and community-level factors, and Model 3 included the outcome variable and both individual and community-level factors.

All analyses were conducted using R software version 4.2.1. DHS Sample weights were applied to correct for the under or over-sampling of different strata during sample selection of the NDHS in accordance with the DHS guidelines [27].

## Results

9,897 married women were included in the spatial and multilevel analysis. Table 1 presents the socio-demographic characteristics of included married women by spousal separation status. Overall, about 66% of women in the sample aged 15–49 years old were living with husbands, while 34% of women were living separately from their husbands (Table 1).

**TABLE 1:** Descriptive statistics of spousal separation by social and economic status. Nepal Demographic and Health Surveys, 2016.

Characteristic	Living together N = 6,450 (weighted %)	Spousal separation N = 3,447 (weighted %)	*p*-value
Individual-level			
Unmet need for FP			<0.001
No	89.60	50.40	
Yes	10.40	49.60	
Duration of separation			<0.001
Living together	100.00	0.00	
Less than 1 year	0.00	50.70	
More than 1 year	0.00	49.30	
Age			<0.001
15–25	20.70	31.10	
26–34	33.80	44.20	
35–49	45.60	24.80	
Female Education			<0.001
Less than primary	42.70	35.80	
Primary	18.10	20.00	
Secondary	27.50	32.60	
Higher	11.60	11.60	
Husband Education			<0.001
Less than primary	31.50	23.90	
Primary	36.80	44.20	
Secondary	12.50	16.00	
Higher	19.10	15.90	
Caste/Ethnicity			<0.001
Brahmin & Chhetri	29.30	29.70	
Dalit	11.90	14.50	
Janajati	30.00	29.80	
Newar	5.60	2.70	
Muslim	4.70	5.90	
Other	18.50	17.40	
Wealth			<0.001
Rich	44.70	36.90%	
Middle	19.40	24.60%	
Poor	35.90	38.50%	
Abortion			>0.9
No	94.90	94.90	
Yes	5.10	5.10	
Number of family			<0.001
Less than 4	16.80	32.60	
4	20.60	18.40	
5	19.50	15.60	
More than 5	43.10	33.30	
Number of children			<0.001
0	9.90	11.20	
1	18.40	27.60	
2	28.70	31.40	
More than 2	43.00	29.80	
District-level			
Residence			0.001
Urban	63.00	57.20	
Rural	37.00	42.80	
Ecology			0.2
Hill	42.80	40.50	
Terai	51.10	54.10	
Mountain	6.10	5.40	
Province			<0.001
Koshi	17.20	16.00	
Madhesh	20.40	25.00	
Bagmati	22.20	14.10	
Gandaki	8.50	11.90	
Lumbini	17.60	17.90	
Karnali	6.00	5.90	
Sudurpashchim	8.20	9.20	
Spousal separation			<0.001
<25%	18.50	6.80	
25%–35%	45.90	40.70	
35%–45%	25.60	33.80	
45%–55%	8.60	15.10	
>55%	1.30	3.60	
Travel time to health facility			<0.001
Less than 30 min	54.80	59.80	
30 min–60 min	24.30	25.60	
More than 60 min	20.90	14.60	

### Spatial Analysis

**FIGURE 1:**
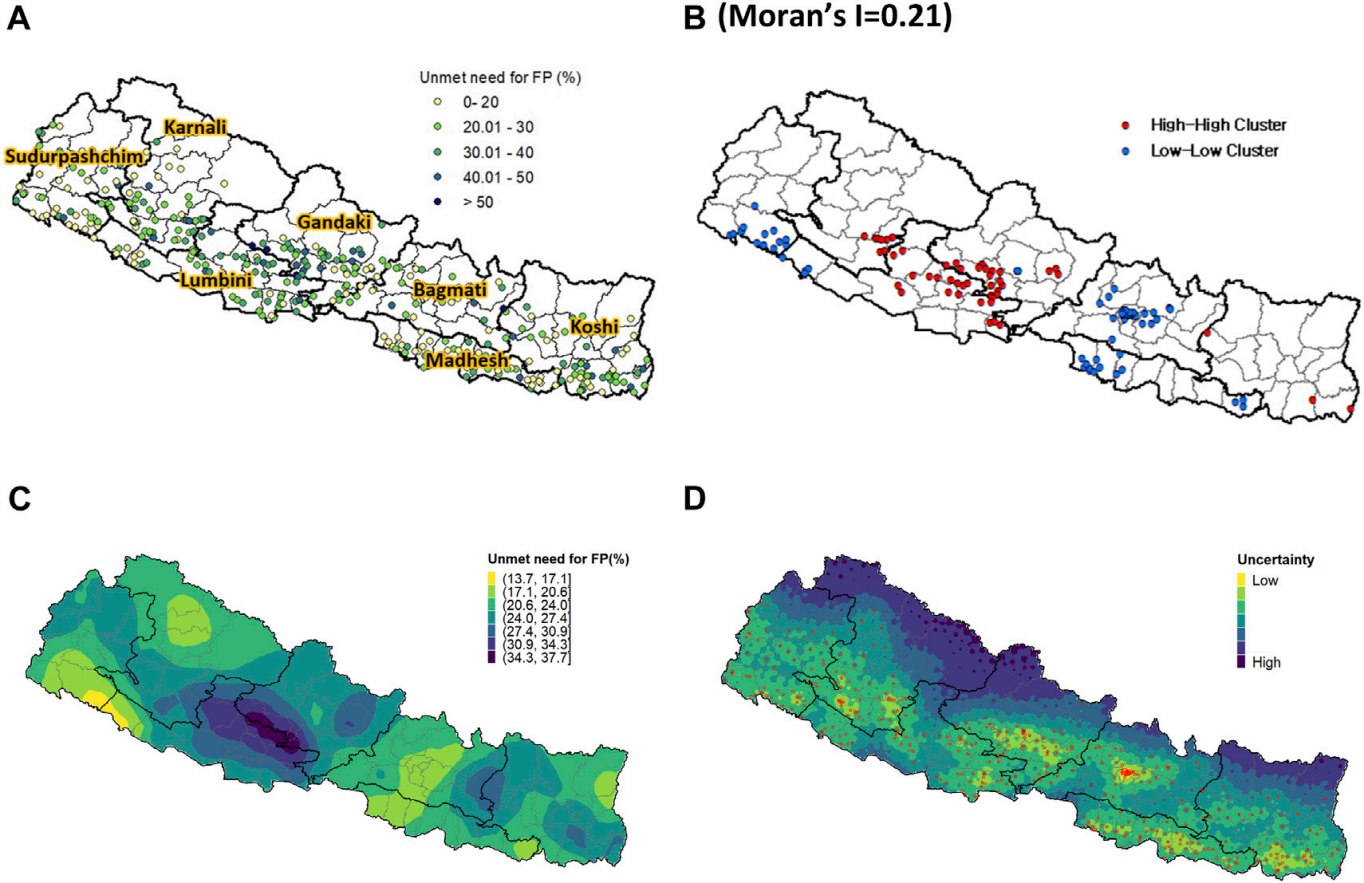
**(A)** Observed unmet need for family planning at the PSU level; **(B)** High-high (HH) and low-low (LL) cluster analysis of UMN; **(C)** Predicted unmet need for family planning from the INLA-SPDE model; **(D)** Uncertainty level. Nepal Demographic and Health Surveys, 2016.

The spatial analysis revealed that the spatial distribution of prevalence of UMN in Nepal significantly varied at the PSU level (Figure 1A) and across the country, with Global Moran’s I value of 0.21. High-High patterns of PSUs were observed around central parts of Nepal (Figure 1B). Low-Low patterns of PSUs were found around the capital and eastern parts of Madhesh and Southern parts of Sudurpashchim.

Similarly, the predicted high-resolution map of UMN showed that central parts of Nepal were predicted as the riskiest areas for UMN, whereas the predicted low-risk areas for UMN were found around the capital in Bagmati, southern parts of Sudurpashchim, and northern parts of Karnali (Figure 1C). The quantified uncertainty associated with the predicted high-resolution map of UMN further showed that relatively higher uncertainty was identified in mountain areas of Nepal where a small number of or no PSUs were selected for the 2016 NDHS (Figure 1D).

**FIGURE 2:**
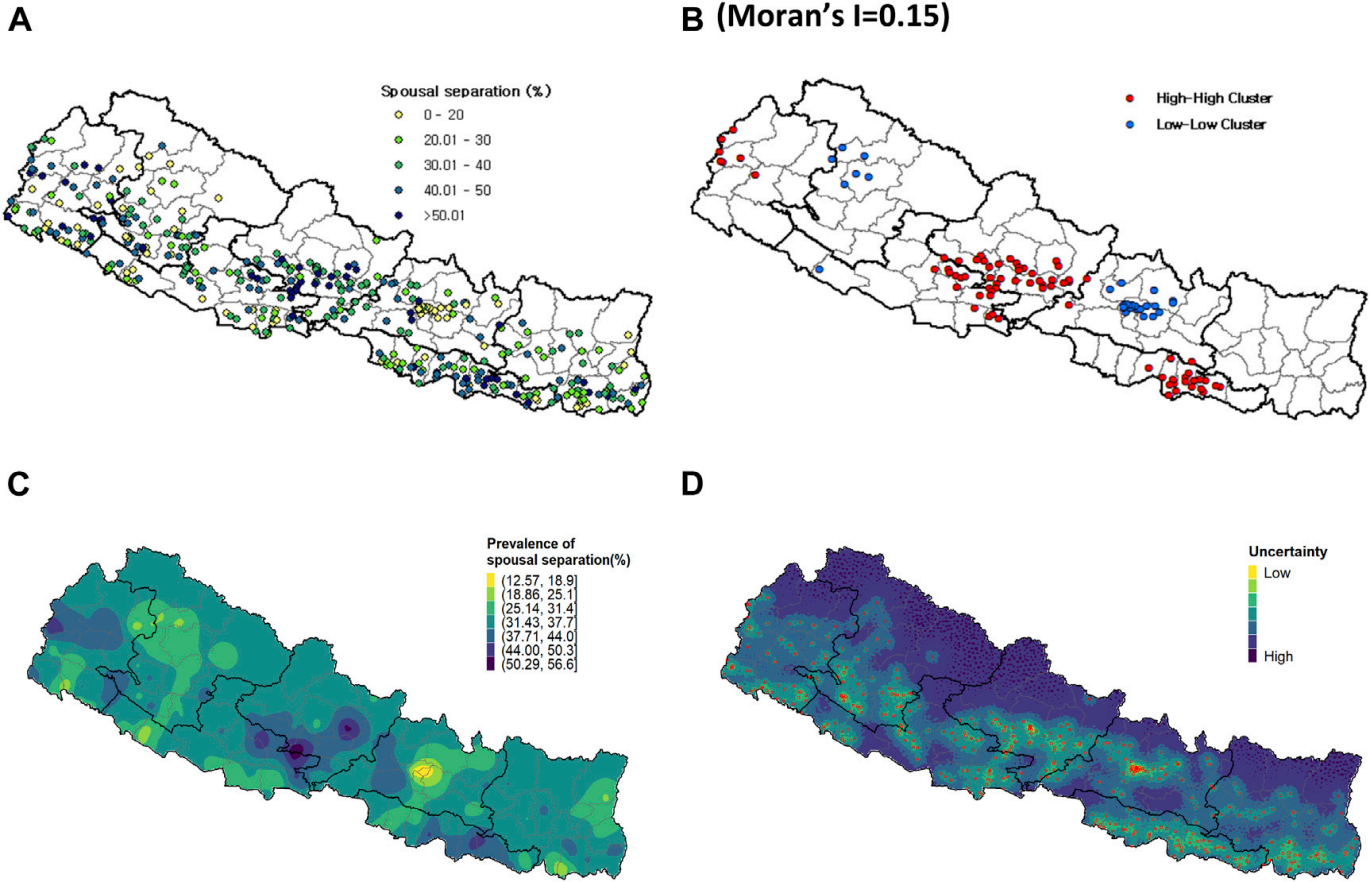
**(A)** Observed spousal separation at the PSU level; **(B)** High-high (HH) and low-low (LL) cluster analysis of spousal separation; **(C)** Predicted spousal separation from the INLA-SPDE model; **(D)** Uncertainty level. Nepal Demographic and Health Surveys, 2016.

In addition, the spatial distribution of percentage of spousal separation in Nepal also varied at the PSU level (Figure 2A) with Global Moran’s I value of 0.15. High-High patterns of PSUs were concentrated around southern parts of Madhesh and central parts of the country (Figure 2B). Low-Low patterns of PSUs were found around the central parts of Bagmati.

The predicted high-resolution map of spousal separation revealed that eastern parts of Lumbini and Gandaki as well as south-eastern parts of Gandaki were identified as the areas most likely to experience high level of spousal separation. On the other hand, the map also indicated that the areas around the capital city of Bagmati, in particular, were identified as areas with a low likelihood of spousal separation (Figure 2C). The quantified uncertainty associated with the predicted high-resolution map of spousal separation exhibited a distribution similar to that of UMN (Figure 2D).

### Multilevel Analysis

The calculated ICC was 10% in the null model, which shows that 10% of the total variability in UMN was attributable to between-district variability, with the remaining 90% attributable to individual differences or unmeasured factors (Table 2). AIC was used for model comparison, and the two-level logistic regression model (Model 3) was selected as the final model (Table 2).

**TABLE 2:** Summary of multilevel logistic regression analysis of unmet need for family planning. Nepal Demographic and Health Surveys, 2016.

	Model 0	Model 1	Model 2	Model 3
Individual-level				
Unmet need for FP				
living with her		0.13 (0.12, 0.15)		0.12 (0.11, 0.13)
staying elsewhere		Ref		Ref
Age				
15–25		Ref		Ref
26–34		0.62 (0.53, 0.73)		0.62 (0.53, 0.73)
35–49		0.39 (0.32, 0.48)		0.39 (0.32, 0.47)
Female Education				
Less than primary		0.80 (0.62, 1.03)		0.87 (0.67, 1.11)
Primary		1.01 (0.79, 1.28)		1.05 (0.82, 1.33)
Secondary		1.21 (0.99, 1.49)		1.24 (1.01, 1.52)
Higher		Ref		Ref
Husband Education				
less than primary		0.98 (0.79, 1.21)		0.97 (0.78, 1.20)
primary		1.24 (1.03, 1.49)		1.21 (0.99, 1.47)
secondary		1.23 (1.00, 1.50)		0.09 (0.97, 1.45)
higher		Ref		Ref
Caste/Ethnicity				
Brahmin & Chhetri		Ref		Ref
Dalit		1.17 (0.97, 1.43)		1.19 (0.97, 1.45)
Janajati		0.96 (0.82, 1.12)		0.94 (0.80, 1.10)
Newar		1.13 (0.84, 1.52)		1.11 (0.83, 1.49)
Muslim		1.27 (0.95, 1.71)		1.33 (0.98, 1.79)
Other		0.82 (0.66, 1.01)		0.92 (0.73, 1.15)
Wealth				
Poor		1.06 (0.90, 1.24)		1.03 (0.88, 1.22)
Middle		0.97 (0.83, 1.14)		0.98 (0.83, 1.15)
Rich		Ref		Ref
Abortion				
No		Ref		Ref
Yes		1.69 (1.34, 2.13)		1.62 (1.29, 2.05)
Number of family				
Less than 4		Ref		Ref
4		1.00 (0.84, 1.19)		1.01 (0.86, 1.20)
5		1.18 (0.99, 1.41)		1.19 (1.00, 1.44)
More than 5		1.20 (1.02, 1.40)		1.22 (1.04, 1.42)
Number of children				
0		Ref		Ref
1		2.19 (1.77, 2.70)		2.24 (1.81, 2.75)
2		2.55 (2.04, 3.21)		2.62 (2.09, 3.29)
More than 2		2.56 (1.98, 3.30)		2.63 (2.03, 3.40)
District-level				
Residence				
Urban		Ref		Ref
Rural		1.04 (0.92, 1.19)		1.03 (0.91, 1.17)
Ecology				
Hill			Ref	Ref
Terai			0.87 (0.74, 1.02)	0.89 (0.72, 1.11)
Mountain			0.89 (0.71, 1.12)	0.92 (0.70, 1.21)
Province				
Koshi			Ref	Ref
Madhesh			0.73 (0.61, 0.87)	0.63 (0.48, 0.83)
Bagmati			0.81 (0.67, 0.98)	0.88 (0.68, 1.13)
Gandaki			0.97 (0.79, 1.20)	0.96 (0.73, 1.26)
Lumbini			1.18 (1.01, 1.39)	1.18 (0.95, 1.47)
Karnali			0.91 (0.69, 1.19)	0.80 (0.57, 1.12)
Sudurpashchim			0.77 (0.61, 0.97)	0.71 (0.53, 0.96)
Spousal separation				
<25%			Ref	Ref
25%–35%			1.56 (1.27, 1.91)	1.09 (0.84, 1.41)
35%–45%			1.99 (1.63, 2.42)	1.13 (0.87, 1.46)
45%–55%			2.29 (1.81, 2.89)	1.22 (0.89, 1.66)
>55%			3.40 (2.31, 5.01)	1.46 (0.89, 2.41)
Travel time to health facility				
Less than 30 min			Ref	
30 min–60 min			0.91 (0.79, 1.06)	0.85 (0.70, 1.03)
More than 60 min			1.21 (1.01, 1.46)	1.30 (1.03, 1.65)
ICC	0.10	0.09	0.04	0.05
AIC	10,283	8,316	9,221	8,203

At the individual level (level 1), six variables were significantly associated with UMN. Married women who were living with husbands had 88% (AOR = 0.12, 95% CI 0.11–0.13) decreased odds of experiencing UMN than married women who were separated from their husbands. The odds of experiencing UMN among married women in the 26–34 and 35–49 age groups decreased by 38% (aOR = 0.62, 95% CI 0.53–0.73) and 61% (aOR = 0.39, 95% CI 0.32–0.47), respectively, compared to married women 25 years old and younger. Compared to women who acquired higher education (greater than secondary level education), women had 1.24 times higher odds of experiencing UMN (aOR = 1.24, 95% CI 1.01–1.52) when they attained secondary education. Moreover, the odds of UMN significantly increased with the number of children. Women who had more than two children had 2.63 times (aOR = 2.63, 95% CI 2.03–3.40) higher odds of experiencing UMN compared with those having no child. Similarly, larger family size was related to higher odds of UMN, as evidenced by 1.22 times (aOR = 1.22, 95% CI 1.04–1.42) higher odds among women living with more than five family members than women living with less than four family members. Married women who had experienced abortion had 1.62 times higher odds of experiencing UMN (aOR = 1.62, 95% CI 1.29–2.05) relative to their counterparts with no experience of abortion.

At the district level (level 2), the odds of experiencing UMN among married women who lived in Madhesh and Sudurpashchim decreased by 37% (aOR = 0.63, 95% CI 0.48–0.83) and 29% (aOR = 0.71, 95% CI 0.53–0.96), respectively, compared with women living in Koshi. Moreover, the odds of experiencing UMN among women residing in districts where the average travel time to health facilities took longer than 1 hour were 1.30 times (aOR = 1.30, 95% CI 1.03–1.65) compared to those living in with districts where the average travel time to health facilities was less than 30 min. Although there was no significant difference in experiencing UMN by district-level prevalence of spousal separation, the odds of UMN increased with higher prevalence of spousal separation at the district level.

## Discussion

While the high prevalence of spousal separation in Nepal affects UMN of married women, previous studies have tended to focus on only individual- and household-level factors. To address the limitations of previous studies, firstly, this study used Bayesian geostatistical approach to show that spousal separation and UMN exhibit similar geographical patterns across Nepal, which is the main novelty of this study. Secondly, this study further contributes to the existing literature on the UMN in high out-migration countries by demonstrating that the UMN among individual couples in the country can be linked to the prevalence of spousal separation in specific areas (diffusion approach) as well as couples’ spousal separation status (adaptation approach).

Firstly, this study found clear spatial patterns where neighbouring locations had similar levels of UMN and spousal separation. Specifically, low prevalence of UMN was observed particularly around the capital city in Bagmati and eastern parts of Madhesh and southern parts of Sudurpashchim. Lower prevalence of UMN could be linked to better family planning service availability around the capital city and the reproductive health service programs around the Nepal-India border in Madhesh and Sudurpashchim, where higher number of migrants living with HIV reside [28]. In contrast, higher prevalence of UMN in central parts of Nepal (Gandaki and Lumbini) could be related to cultural contexts of different ethnic groups. For instance, the Magar community, whose birth rates are higher than the national average, reside in central parts of Nepal [29]. While ethnicity was not significantly associated with UMN in the regression analysis, this result may be due to grouping different ethnic groups together.

Secondly, at the individual-level, married women separated from their migrant husbands were significantly more likely to experience UMN, consistent with previous studies on the association between spousal separation and UMN in Nepal [4, 7, 21] and India [8, 10]. Also consistent with previous studies, our findings indicated that education level of both women and their husbands were important factors of UMN [11]. Husband’s education level can influence UMN through various mechanisms, such as change in husbands’ attitudes and perception towards contraceptive use [20, 30]. A previous study in Pakistan found that when husbands had primary education compared to no education, UMN was higher, while the positive association was not found for husbands with secondary education or higher [29]. In the same study, UMN was higher when the husband was the decision-maker for not using contraceptives and when the reason for not using contraceptives was that the husband opposed. As spousal separation hinders partner communication about family planning, husband’s influence in contraceptive use may be another reason UMN is higher among separated couples [31]. These findings imply that the UMN among married women can be explained by a response to socioeconomic circumstances, which is consistent with the adaptation view. At the district-level, while not significant, the odds of UMN increased in a linear manner as the prevalence of spousal separation at the district level increased, even after individual- and household-level variables including spousal separation at the individual level were controlled. A similar result was found in India where a clear effect of the migratory environment on contraceptive use and access to family planning services was observed between high male out-migration and low male out-migration areas [8]. Potential reasons for the contextual effect may include barriers due to a lack of access to family planning services and particular social norms in areas with high rates of spousal separation. Mukherjee and Mahapatra [9] found that community healthcare workers perceived wives of migrant workers not as users of contraceptives and therefore family planning outreach was poor in areas with high male out-migration [9]. Districts where spousal separation is more common in Nepal may have strong social and community norms restricting women’s access to reproductive health services in absence of their husbands [18]. Women may experience greater restrictions if they reside with their mothers-in-law who have significant influence in contraceptive use of daughters-in-law in South Asia [10, 32]. These findings suggest that UMN among married women in Nepal could be influenced by both individual and district-level social and cultural contexts, which is consistent with the diffusionist view. Overall, these results, derived from analyses at both the individual and district levels, suggest that the UNM in Nepal should be explained by encompassing both adaptation and diffusion perspectives.

These findings have implications for re-visiting the measurement of UMN and reproductive health policy addressing the UMN for both married couples living apart and in areas with high spousal separation. In terms of implications for measuring the UMN, previous studies report that women were finding out about husbands’ return a month in advance on average and waited until husbands’ arrival to use contraceptives [8, 9]. In addition, social perception created barriers for women to accessing reproductive health services in absence of their husbands, even if the husbands are about to return [18, 33]. In this context, a time gap between husbands’ return and initiation of family planning methods may occur [9, 18]. While it cannot be assumed that a couple is completely sexually inactive during separation, women not cohabiting with their spouses rarely use or report use of contraceptives. Since UMN is calculated as the period when married women who do not intend to have more children are not using methods of contraception, the period of spousal separation due to migration is calculated as UMN. Calculation of overall UMN without stratifying for spousal separation can explain the stagnant progress in reducing UMN in Nepal [4, 21]. Such calculation can also miss the actual timepoints of UMN with migrant husbands’ return. Findings of this study suggests that to accurately measure UMN in high male out-migration contexts such as Nepal, a more tailored approach is necessary. In terms of policy implications, it is important to recognize that married women separated from their spouses and women residing in areas with a high prevalence of spousal separation may have distinct needs for family planning services. Firstly, it is important to disaggregate UMN by spousal separation status as sexual behaviour, family planning intentions, and access to family planning services are different for separated spouses. Calculating the overall UMN without considering spousal separation, especially in countries with high levels of out-migration of young people such as Nepal, may be less useful to understand UMN. UMN is used as one of the main indicators to measure progress in achieving Sustainable Development Goals (SDGs) on universal access to sexual and reproductive healthcare services (Goal 3.7) and reproductive rights (Goal 5.6). Reflecting the migration context in calculating UMN will enable the Nepalese government to gauge the progress made through recent efforts such as the Right to Safe motherhood and Reproductive Health Act, 2018. Secondly, spousal separation needs to be a key factor considered for audience segmentation to adapt family planning services and programs accordingly. Spouses of migrant workers, especially in high out-migration areas, may be neglected from community efforts to raise awareness of modern contraceptives and make them more accessible. A tailored outreach can include messages focused on effective use of contraceptive methods for couples who meet with intervals. Interventions can also target social norms around women accessing family planning services while they are separated physically from their spouses. To lower women’s barriers to access in the absence of their husbands, engagement of migrant husbands and mothers-in-law would be beneficial. An evidence-based intervention to reduce UMN among separated spouses might target partner communication about intimate topics [31].

This study uses rigorous spatial modelling techniques to demonstrate geographical patterns of UMN associated with spousal separation across Nepal. A multilevel regression model that incorporates individual, household, and community levels complement the results from the spatial analyses. However, this study has several limitations. Firstly, this study primarily focuses on the Nepalese contexts with relatively small sample size, which may limit the generalizability of the findings to other country contexts. Uncertainty across the modelled surface can result from small sample sizes at each cluster with approximately 25–35 women from each cluster. As a result, samples are less likely to accurately represent the population of each cluster, thereby increasing the risk of misleading estimates. Although we used spatial modelling to mitigate the risk of extreme high or low estimates by borrowing information from neighbouring areas, it is important to consider the uncertainty map (Figures 2D) when interpreting the modelled surface of the prevalence of UMN and spousal separation throughout the country. Secondly, while spousal separation was interpreted considering high prevalence of labor out-migration in Nepal, we could not directly verify the reason of spousal separation as well as detailed circumstances of separation such as the destination of the migrating spouse or their frequency of return from the data. While migration destination can influence the interval at which spouses return or visit home, which affect sexual behaviors and UMN, this limitation prevented us from examining differences by destination. The lack of specifics related to husbands’ migration also limit the generalizability of the results to other contexts of high out-migration, where different migratory patterns may exist. Follow-up studies with more background information about husbands’ migration will provide further details useful to tailor family planning services for separated spouses. Similar to Mahapatra and Saggurti’s study [8], follow-up studies should also include perceptions of community healthcare workers and their outreach to wives of migrant workers. Thirdly, couple-level variables including spousal communication can significantly influence UMN [11, 34]. However, dyadic variables were not available in the NDHS dataset. Since this study used women’s report of spousal separation, the results are generalizable to households where husbands migrate for work. Future studies should explore how UMN is experienced in households where wives migrate, as seen in Koshi and Bagmati [12]. Lastly, we found that the 2022 NDHS was recently released in July 2023. Given our understanding that the COVID-19 pandemic significantly influenced spousal separation status in Nepal, it is imperative to carefully compare the UMN-associated spousal separation between before and during the pandemic. Therefore, we commit to conducting a comparative study of UMN-associated spousal separation before and during the COVID-19 period for our next research.

### Conclusion

Analysing the Nepal DHS data, this study found geographical patterns in unmet need for family planning (UMN) in Nepal. This variation in UMN was associated with spousal separation not only at the individual level but also at the community level. To address UMN in Nepal, which remained consistently high over the past decade, individual and local context-specific family planning programmes should be tailored to wives living separated from their husbands.
