# Supplementary material for: Unmet Need for Family Planning and Spousal Separation in Nepal: A Spatial and Multilevel Analysis
Source: Int J Public Health. 2023 Dec 7;68:1606395. doi: 10.3389/ijph.2023.1606395 (PMC10733444; doi:10.3389/ijph.2023.1606395)
Supplement: Supplementary file 1 [file DataSheet1.PDF]

Supplementary Appendix: Details of the geostatistical modelling

To predict the prevalence of unmet need for family planning (UMN) and spousal separation of married women across Nepal. We first assumed that the prevalence of UMN and spousal separation are a spatially continuous across Nepal, which can be modelled using a Gaussian random field (GRF). We then used the Bayesian geostatistical modelling framework. Conditional on the true prevalence $P(x_i)$ of UMN or spousal separation at location $x_i, i = 1, \dots, n$, we assumed that the number of cases Y_i out of N_i women sampled follow a binomial distribution: $Y_i|P(x_i) \sim Binomial\ (N_i, P(x_i))$.

Then, mean predicted prevalence of UMN and percentage of spousal separation was modelled via a logit link function to a linear predictor defined as:

$$logit\big(P(x_i)\big) = a + S(x_i)$$

where α is the intercept and $S(\cdot)$ is a spatial random effect modelled using a zero-mean Gaussian Markov random field with a Matérn covariance function, given by,

$$Cov(S(x_i)\ , S(x_j)) = \frac{\sigma^2}{2^{v-1}\Gamma(v)}(k||\ x_i - x_j||)^v K_v(k||\ x_i - x_j||)$$

where σ^2 is the spatial process variance. The term $K_v(\cdot)$ denotes the modified Bessel function of second kind and order $v > 0$, which measures the degree of smoothness. Conversely, $\kappa > 0$ is a scaling parameter related to the spatial range $r = \frac{\sqrt{8v}}{k}$ that is the distance at which the spatial correlation becomes almost null (see example by Lindgren et al. (2011) for a detailed description).

Then, we applied the stochastic partial differential equation method (SPDE) using the R-INLA package to fit a spatial model and make predictions for the prevalence of UMN and spousal separation at unsampled locations (Lindgren and Rue 2015). The Matérn covariance matrix represents a Gaussian random field (GRF) in the continuous domain SPDE equation proposed by Whittle (1963):

$$(k^2 - \Delta)^{\frac{a}{2}}(\tau x(s) = W(s)$$

Here, x(s) denotes the GRF and W(s) represents a Gaussian spatial white noise process. The parameter α controls the smoothness of the GRF, τ controls the variance, and κ>0 serves as a scale parameter. Δ refers to the Laplacian defined $\sum_{i=1}^{d} \frac{\partial^2}{\partial x_i^2}$ where d denotes the dimension of the spatial domain D.

The parameters of the Matérn covariance function and the SPDE are interconnected as follows. The smoothness parameter ν of the Matérn covariance function is related to the SPDE by the equation:

$$v = a - \frac{d}{2,}$$

For d=2 and ν=1/2, which corresponds to the exponential covariance function. The default value for α is 2, and we utilised the default value for our analysis.

For these analyses, we assumed an uninformative prior distribution on model parameters to allow the data to drive model results. As no previous literature or data exist to inform our expectations of the spatial distribution of UMN and spousal separation across Nepal, there was insufficient evidence to otherwise inform a prior distribution.

The Bayesian geostatistical model analysis was implemented using a stochastic partial differential equations (SPDE) approach in the INLA algorithm as applied in the R-INLA package (Rue et al., 2009). This algorithm provides an effective estimation and spatial prediction strategy for spatial data by specifying a spatial data process as well as a spatial covariance function depending on locations and time points at which outcome and covariate data are collected (Rue et al., 2009). The SPDE allows us to define a grid on spatial data by creating a constrained refined Delaunay triangulation (usually called mesh) over the study region. With this approach, observations are treated as initial vertices for the triangulation (**Figure 1**). As opposed to the regular grid, this approach is denser in regions where there are more observations and consequently generates more information. This approach also saves computing time because prediction locations are typically much fewer than those in a regular grid. To show uncertainty related to the prediction, we presented the spatial distribution of credible intervals by calculating the differences between 5% and 95% percentiles of the predicted UMN and spousal separation across the country.

Figure 1. Mesh Creation including locations of primary sampling units in the 2016 NDHS

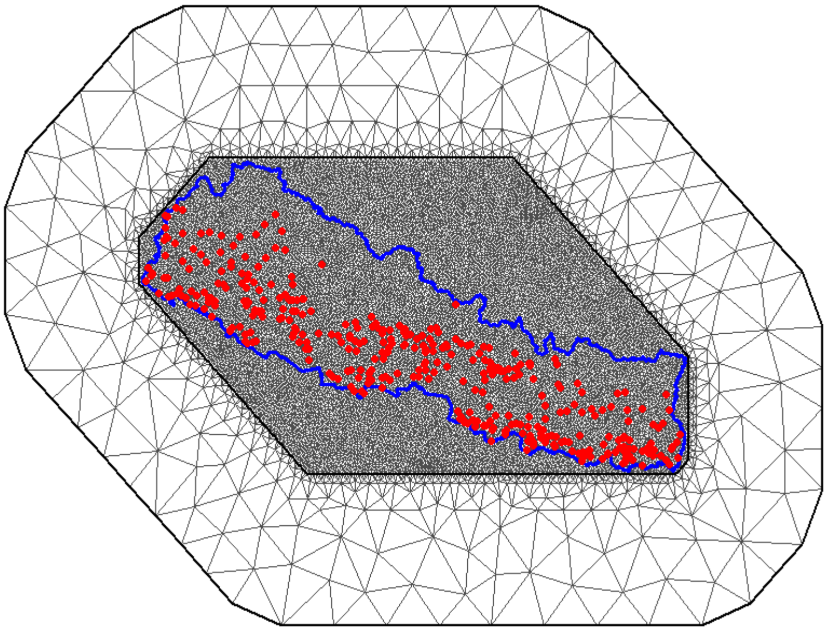

Reference

Lindgren, F., Rue, H., & Lindström, J. (2011). An explicit link between Gaussian fields and Gaussian Markov random fields: the stochastic partial differential equation approach. Journal of the Royal Statistical Society: Series B (Statistical Methodology), 73(4), 423-498.

Lindgren, F., & Rue, H. (2015). Bayesian spatial modelling with R-INLA. Journal of statistical software, 63(19).

Whittle, P. (1963). On the fitting of multivariate autoregressions, and the approximate canonical factorization of a spectral density matrix. Biometrika, 50(1-2), 129-134.
